# Prevalence and Symmetry of Positional Anomalies in Second Permanent Molars: Study of Romanian Patients

**DOI:** 10.3390/pediatric16040097

**Published:** 2024-12-10

**Authors:** Rahela Tabita Moca, Abel Emanuel Moca, Mihai Juncar

**Affiliations:** 1 Doctoral School of Biomedical Sciences, University of Oradea, 410087 Oradea, Romania; rahelamoca@gmail.com; 2 Department of Dentistry, Faculty of Medicine and Pharmacy, University of Oradea, 410073 Oradea, Romania; mihaijuncar@gmail.com

**Keywords:** positional anomalies, second permanent molars, Romanian patients

## Abstract

Background/Objectives: This study aimed to investigate the prevalence and characteristics of positional anomalies in second permanent molars among Romanian patients. These molars play a crucial role in occlusion but can exhibit positional issues such as tilting, rotation, infraocclusion, and impaction. Methods: This retrospective study examined the digital models of 103 patients aged 12–40, which were obtained by using the Medit i500 intraoral scanner. Positional anomalies were categorized by tilting, rotation, infraocclusion, and impaction. Results: The results showed a high prevalence of anomalies, particularly infraocclusion and buccal tilting in upper molars and oral tilting and mesio-buccal rotations in lower molars. The significant symmetry of anomalies within the same dental arch was noted. Gender and malocclusion type did not significantly influence anomaly frequency. Conclusions: The findings emphasize the need for the vigilant monitoring of second permanent molars to maintain functional occlusion and suggest potential common etiological factors within dental arches. Despite this study’s limitations, including sample size and retrospective design, this study underscores the clinical importance of the early detection and management of molar anomalies. Future research should expand on these findings, considering genetic and environmental influences on dental development.

## 1. Introduction

Dental occlusion encompasses more than the contact between masticatory dental surfaces and opposing teeth and can be more comprehensively defined as the coordinated functional interaction among various cell populations that constitute the masticatory system, involving cells that differentiate, model, remodel, fail, and repair [1]. Occlusion is a fundamental issue in dental medicine and has been extensively studied in the specialized literature to understand its physiology and its relationship with the stomatognathic system [2]. Although it was previously hypothesized that occlusal problems could lead to temporomandibular joint disorders [3], recent studies and literature data suggest that occlusion plays a minimal role as a primary factor in the occurrence of temporomandibular disorders [4]. However, occlusion can be adversely affected by temporomandibular disease [5]. While incisors, canines, and first permanent molars play a significant role in defining occlusion ratios in permanent dentition [6], other teeth such as premolars [7], second permanent molars [8], and third molars [9] also influence occlusion.

Second permanent molars play a crucial role in completing occlusion and determining a correct vertical occlusal dimension, as well as establishing a proper relationship between the condyle and the glenoid fossa [10]. These teeth are typically the last to erupt, with the exception of third molars, usually emerging around the age of 12 [11]. Although they generally erupt without significant issues, there are instances where eruption anomalies occur, such as ectopic eruption, impaction, or the primary failure of eruption [12,13,14]. Eruption anomalies of second permanent molars represent a significant area of interest in pediatric dentistry and orthodontics due to their implications for oral health and development. These eruption anomalies, which may include delayed eruption, impaction, or ectopic positioning, can lead to complications such as crowding, malocclusion, and increased susceptibility to caries and periodontal disease [15,16,17,18,19]. While their etiology is multifactorial, involving genetic, systemic, and local factors, their prevalence and clinical significance demand attention from both researchers and practitioners [20]. Positional anomalies like rotations and tilting can affect permanent molars, negatively impacting normal occlusal relationships [15]. Furthermore, the extraction of the first permanent molar after the eruption of the second permanent molar can cause the mesial tilting of the second permanent molar [15]. From a therapeutic perspective, second permanent molars present a challenge for dentists, regardless of their specialization, and are often overlooked by specialists [17]. Positional anomalies can also be associated with the development of harmful habits such as mouth breathing, finger sucking, atypical swallowing, or the interposition of objects between the teeth [18]. Moreover, patients with periodontal disease may exhibit positional anomalies, especially since all types of dental malpositions can lead to the formation of periodontal pockets on the mesial surfaces of the affected teeth [19].

Eruption anomalies of second permanent molars are not uncommon. Studies have reported that approximately 4.3% of children exhibit delayed eruption [21], with 2.3% presenting impactions or other anomalies [22]. These anomalies vary across populations and are influenced by factors such as age, gender, and underlying systemic conditions [20]. For instance, ectopic eruption has been noted more frequently in patients diagnosed with cleidocranial dysplasia [23]. Table 1 summarizes the most common anomalies of the second permanent molar and their reported frequencies.

In Romania, studies investigating the prevalence of positional anomalies in second permanent molars are lacking, a deficiency similarly observed across Europe. Nonetheless, these teeth play a crucial role in achieving functional occlusion [17]. Given the significance of the second permanent molar and the paucity of the specialized literature addressing the positional anomalies frequently associated with these teeth, the authors deemed it essential to examine this aspect.

Consequently, the purpose of this study was to investigate the positional anomalies of second permanent molars in a sample of patients from Romania. This study also aimed to explore the influence of gender and type of malocclusion on these positional anomalies and to examine the potential symmetry of anomalies within the same dental arch (upper and lower) and within the same hemiface (right or left). We hypothesized that positional anomalies are a common occurrence in second permanent molars among the Romanian population, that the symmetry of these anomalies exists within the same dental arch and hemiface, and that gender and malocclusion type may influence the frequency of positional anomalies in these molars.

## 2. Materials and Methods

### 2.1. Ethical Considerations

This study adhered to the principles of the Declaration of Helsinki and its subsequent amendments. Ethical approval was granted by the Ethics Committee of the Faculty of Medicine and Pharmacy, University of Oradea (IRB No. CEFMF/02, issued on 30 September 2022).

### 2.2. Sample Selection and Examination of Positional Anomalies

This retrospective study analyzed digital study models (scans) from a sample of patients in Oradea, Romania. The scans were collected from patients at a dental clinic in Oradea and were generated from digital impressions taken during initial consultations, which were deemed essential for accurate diagnosis at the time of presentation. The digital impressions were captured using the Medit i500 intraoral scanner (Medit Corp., Seoul, Republic of Korea). For minor patients, informed consent was obtained from their parents or legal guardians, permitting the use of these digital study models in future scientific research. Adult patients provided their own informed consent. This study included patients who first presented to the dental clinic between 1 September 2022 and 1 May 2024.

Patients included in this study met the following criteria: aged 12 or above, with complete permanent dentition (excluding wisdom teeth), and requiring dental treatment. Exclusion criteria comprised patients with extractions or the agenesis of permanent teeth, previous mobile or fixed orthodontic treatment, a significant coronal destruction of second permanent molars, inadequate scans preventing the proper visualization of second permanent molars, the presence of dental crowns on second permanent molars, local or systemic diseases affecting dento-facial growth and development, and incomplete or insufficient patient records. Table 2 summarizes the inclusion and exclusion criteria for this study.

**Figure 1:**
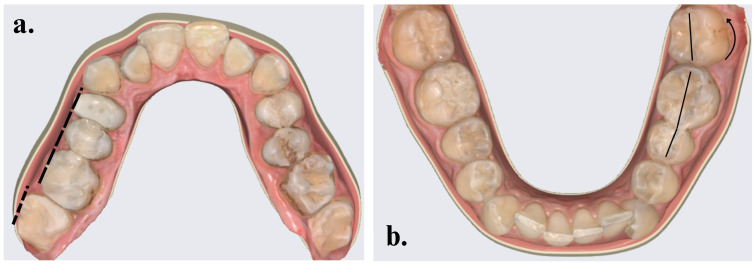
Positional changes in second permanent molars: (**a**) BT of tooth 1.7; (**b**) MBR of tooth 3.7.

The positions of the four second permanent molars were examined: the upper right second permanent molar (1.7), upper left second permanent molar (2.7), lower left second permanent molar (3.7), and lower right second permanent molar (4.7). Axial changes were categorized as the buccal tilting (BT—tilting towards the buccal side), oral tilting (OT—tilting towards the oral side), mesial tilting (MT—tilting towards the mesial side), or distal tilting (DT—tilting towards the distal side) of the dental crown, which were visually assessed by two independent observers. Each observer used a standardized evaluation form that outlined the specific criteria for categorizing each type of tilting and rotation. To minimize subjectivity, the criteria were established based on clearly defined angular thresholds for tilting and rotations, ensuring consistency in classification across evaluations. Additionally, rotational changes were categorized as mesio-buccal rotations (MBR—mesial face rotated towards the buccal side) or disto-buccal rotations (DBR—distal face rotated towards the buccal side), based on predefined rotation degrees. Any other positional anomalies, such as infraocclusion (IO) and impaction (I), were noted (Figure 1).

The patient files also contained information regarding the initial diagnosis of malocclusion according to Angle’s classification (Class I, Class II subdivision 1, Class II subdivision 2, Class III). Angle’s classification was chosen because it is one of the most widely used and recognized systems for categorizing malocclusions based on the position of the first molars and the alignment of the dental arches. This classification provides a standardized method for diagnosing and comparing different types of malocclusion, which is essential for understanding the initial dental conditions of the patients and for ensuring consistency across the patient records. By using this system, we aimed to categorize patients’ malocclusions in a manner that facilitates comparison with the existing literature and clinical standards.

The examination of positional changes on the digital study models was initially conducted by one investigator (R.T.M.) and subsequently verified by another investigator (A.E.M.). The assessment was conducted in a double-blind manner, where neither observer had access to the other’s evaluations during the initial assessment phase. In cases where discrepancies occurred between the two observers’ evaluations, the data were reviewed, and a consensus was reached through discussion. If a consensus could not be reached, a third independent expert observer was consulted to resolve the inconsistency. The inter-rater reliability was 97%, indicating very good agreement for the diagnosis of positional dental anomalies.

### 2.3. Statistical Analysis

Statistical analysis was performed using IBM SPSS Statistics 25 (IBM Corp., Chicago, IL, USA) and Microsoft Office Excel/Word 2021 (Microsoft Corp., Redmond, WA, USA). Qualitative variables were presented as absolute frequencies or percentages. Fisher’s Exact tests were employed to examine differences between independent qualitative variables. Quantitative variables were described using means with standard deviations or medians with interpercentile intervals, with their distribution assessed using the Shapiro–Wilk test. For quantitative variables exhibiting non-parametric distribution, between-group comparisons were conducted using the Mann–Whitney U test. A significance level of *p* < 0.05 was considered statistically significant.

## 3. Results

### 3.1. Sample Characteristics

**Figure 2:**
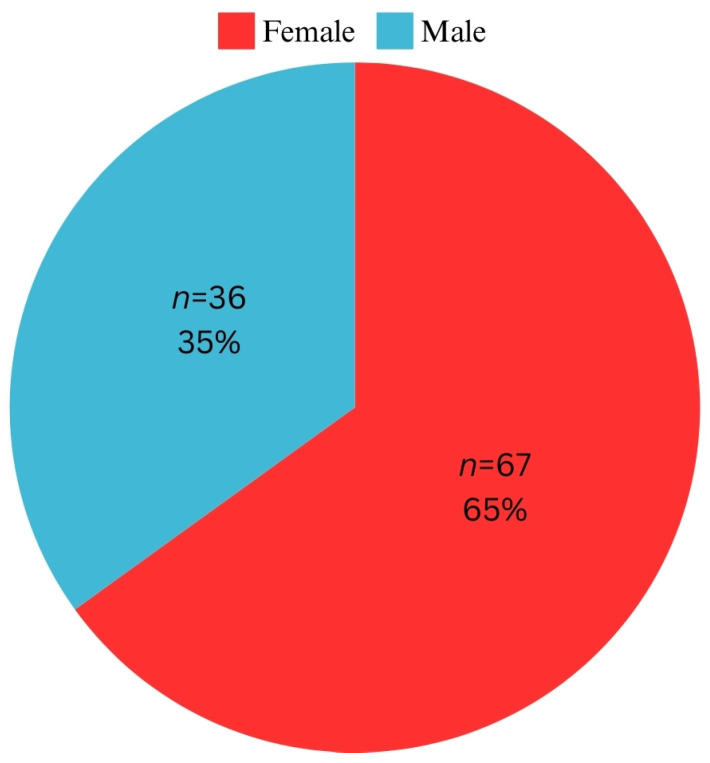
Distribution of patients related to gender.

**Figure 3:**
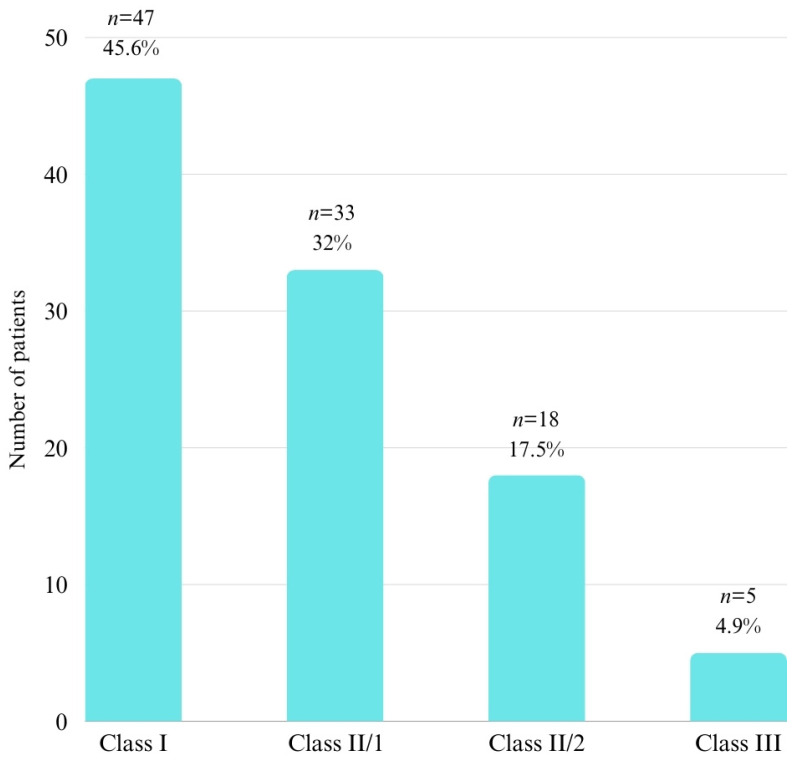
Distribution of patients related to malocclusion.

After applying the inclusion and exclusion criteria, 103 patients remained in this study, the majority of whom were female (*n* = 67, 65%) (Figure 2). Most patients had an initial diagnosis of Class I malocclusion (*n* = 47, 45.6%) (Figure 3). The average age of the patients was 18.81 ± 6.25 years, with a minimum age of 12 years and a maximum age of 40 years.

### 3.2. Positional Anomalies at the Level of the Second Permanent Molars

**Figure 4:**
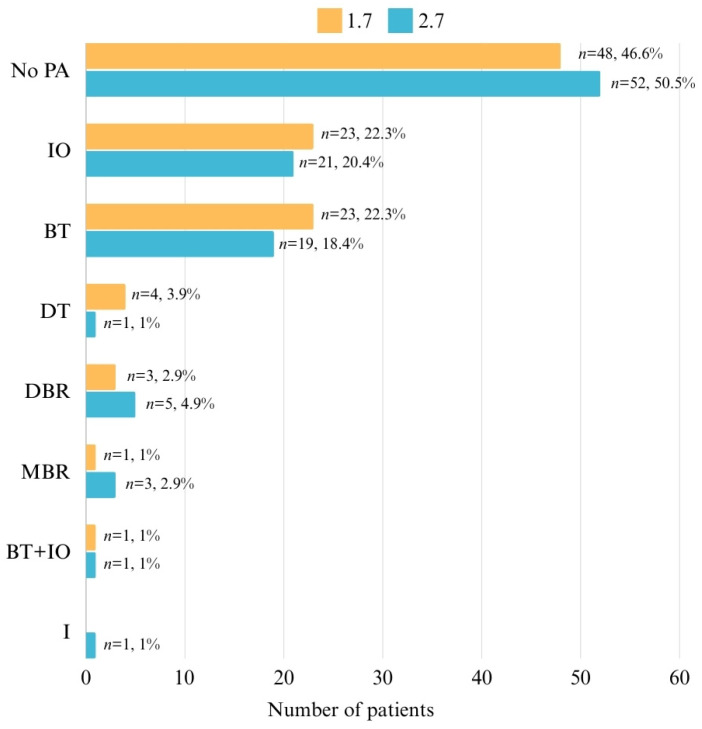
Distribution of patients related to positional anomalies at 1.7 and 2.7.

The data in Figure 4 represent the distribution of patients with positional anomalies at 1.7 and 2.7. Positional anomalies at 1.7 were present in 54.4% of patients, with the most frequent anomalies being IO (22.3%) and BT (22.3%). Similarly, 49.5% of patients had positional anomalies at 2.7, with the most frequent anomalies being IO (20.4%) and BT (18.4%).

**Figure 5:**
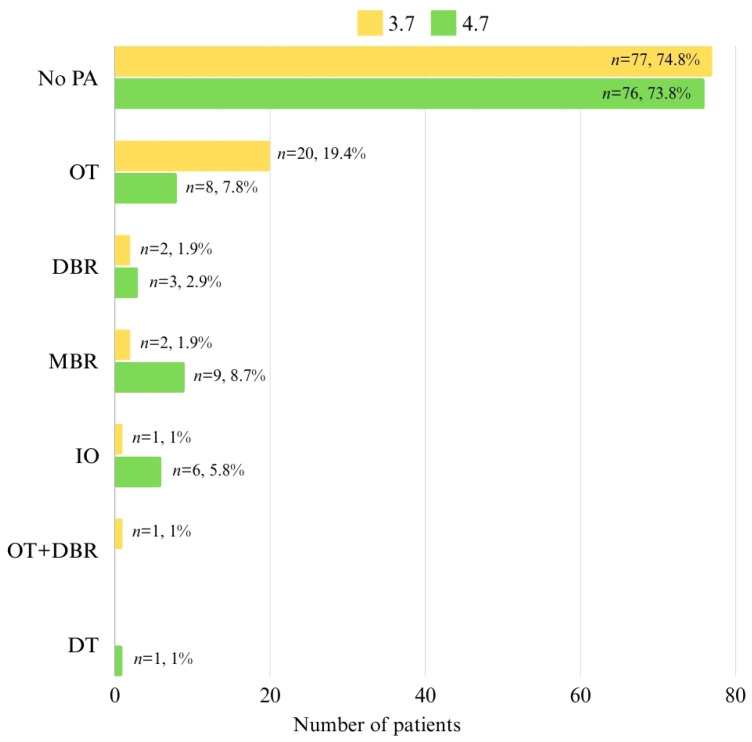
Distribution of patients related to positional anomalies at 3.7 and 4.7.

The data in Figure 5 represent the distribution of patients with positional anomalies at 3.7 and 4.7. Positional anomalies at 3.7 were present in 25.2% of patients, with the most frequent anomalies being OT (19.4%). Similarly, 26.2% of patients had positional anomalies at 4.7, with the most frequent anomalies being MBR (8.7%) and OT (7.8%).

Regarding the symmetry of positional anomalies in the molars located on the upper arch (1.7 and 2.7), it was observed that the differences between the groups were statistically significant according to the Fisher test (*p* < 0.001). Patients with positional anomalies at 1.7 were significantly more likely to have positional anomalies at 2.7 (82.4% vs. 25%). Similarly, for the molars on the lower arch (3.7 and 4.7), the differences between the groups were statistically significant according to the Fisher test (*p* = 0.040). Patients with positional anomalies at 3.7 were significantly more likely to have positional anomalies at 4.7 (40.7% vs. 19.7%) (Table 3).

Regarding the symmetry of positional anomalies for the molars located on the same hemiface (left or right), it was observed that, for molars on the right side (1.7 and 4.7), as well as for molars on the left side (2.7 and 3.7), the differences between groups were not statistically significant according to Fisher’s test. Therefore, there was no significant association between the positional anomalies at these locations (Table 4).

### 3.3. The Influence of Gender and Type of Malocclusion on Positional Anomalies for Second Permanent Molars

It was observed that gender had no influence on positional anomalies in the studied group. For all four investigated molars, the differences between groups were not statistically significant according to the Fisher test. Consequently, the frequency of positional anomalies at 1.7, 2.7, 3.7, and 4.7 was not significantly different based on the gender of the patients (Table 5).

The data in Table 6 represent the distribution of patients according to the type of malocclusion and positional anomalies at 1.7, 2.7, 3.7, and 4.7. The differences between groups were not statistically significant according to the Fisher test, indicating that the frequency of positional anomalies for these four molars did not significantly vary based on the type of malocclusion.

## 4. Discussion

The present study aimed to investigate the positional anomalies of second permanent molars in a sample of patients from Romania. This study analyzed the influence of gender and type of malocclusion on these anomalies and examined their symmetry within the same dental arch and hemiface. To date, no studies have been identified from Romania that address this subject within a patient sample. The only study identified pertained to numerical anomalies, presenting a series of clinical cases diagnosing the congenital absence of permanent second molars [25]. The uniqueness of the present study, which addressed the issue of the positional anomalies of second permanent molars, is therefore an important element of originality.

The relatively small number of participants in the final sample can be explained by the difficulty in identifying patients without extractions and agenesis in the permanent dentition. The extraction of the first permanent molar, which directly impacts the position of the second permanent molar [26], frequently occurs in childhood and adolescence [27]. Additionally, the extraction of other permanent teeth has a relatively high prevalence in the Romanian population, with the World Health Organization (WHO) reporting edentulism in individuals over 20 years of age at 12.4% [28]. The most common causes for the extraction of permanent teeth are complications from dental caries and periodontal disease, although extractions for orthodontic purposes are also notable [29]. Under these conditions, identifying a sample that met the inclusion and exclusion criteria was challenging.

Our study revealed that the positional anomalies of second permanent molars are quite prevalent in the studied population. Specifically, 54.4% of patients exhibited anomalies in the upper right second permanent molar (1.7) and 49.5% in the upper left second permanent molar (2.7), IO and BT being the most common anomalies. In the lower molars, 25.2% of patients had anomalies in the lower left second permanent molar (3.7) and 26.2% in the lower right second permanent molar (4.7), OT and MBR being the most frequent. Additionally, significant symmetry was observed for anomalies within the same dental arch but not within the same hemiface. Infraocclusion is more frequently encountered in deciduous molars, but it can also affect second permanent molars, with treatment options ranging from orthodontic interventions to extraction in severe cases [30].

The findings of our study align with previous research that emphasizes the importance of second permanent molars in maintaining functional occlusion and the vertical dimension of occlusion [10,31]. La Monaca et al. (2019) highlighted the importance of the early diagnosis of the failed or delayed eruption of first and second permanent molars in achieving optimal therapeutic outcomes from all perspectives [10]. Similarly, Cernei et al. (2015) presented an innovative method for uprighting a second permanent molar that had mesially tipped following the extraction of a first permanent molar. Achieving the optimal position of the second permanent molar was essential for establishing proper occlusion [31].

Studies in the literature that have investigated positional anomalies affecting these molars, such as rotations, tilting, and infraocclusion, which can disrupt normal occlusal relationships, are almost nonexistent [15]. Only one similar study was identified, conducted by Viganó et al. (2016), in which the authors analyzed the average rotations of first permanent molars in patients with Angle Class I, II, or III malocclusions [15]. However, no studies were identified that investigated these rotational changes in second permanent molars. Beyond this innovative aspect, our study uniquely highlights the significant occurrence of infraocclusion and buccal tilting in upper molars and the prominence of oral tilting and mesio-buccal rotations in lower molars.

Interestingly, this study did not find statistically significant results regarding the influence of Angle malocclusions on the positional anomalies of second permanent molars. This outcome could be attributed to the relatively small sample size and the unequal distribution of different malocclusion types. Globally, the prevalence of various Angle malocclusion types also shows variability, with Class I accounting for 72.74%, Class II for 23.11%, and Class III for 3.98% [32]. In Romania, a recent study indicated that while Angle Class I malocclusion is the most prevalent (48.78%), Angle Class II malocclusions also demonstrate a significant prevalence (45.85%) [33]. The distribution observed in this study aligns closely with findings from other studies conducted in Romania. However, no additional studies were identified to facilitate broader comparisons.

Another critical point to highlight is that although patients with fully erupted third molars were excluded from this study, unerupted third molars might still have been present within the maxilla and would only have been detectable on radiographic imaging. Third molars can exert eruptive pressure on permanent teeth, potentially leading to minor changes even after orthodontic treatment [34]. Furthermore, this eruptive pressure may negatively impact permanent second molars by increasing the risk of root resorption and bone loss on their distal surfaces [35].

These results have important clinical implications. The high prevalence of positional anomalies in second permanent molars suggests that these teeth should be closely monitored during routine dental examinations. Our study investigated the anomalies of the second permanent molars because, despite their critical role in maintaining functional occlusion and vertical dimension, they are less studied compared to canines or first molars. Their late eruption and susceptibility to positional disturbances such as infraocclusion, tilting, and rotations highlight a gap in the literature that this study sought to address, contributing to an improved understanding and management of these issues in clinical practice. The early detection and management of such anomalies are crucial to prevent potential complications in occlusion and masticatory function. Additionally, the significant symmetry of anomalies within the same dental arch implies a potential common etiological factor that may need to be addressed in dental treatments and orthodontic planning.

Regarding the hypotheses, this study confirms the first hypothesis, demonstrating a high prevalence of positional anomalies in second permanent molars among the Romanian population. The second hypothesis, concerning the symmetry of anomalies within the same dental arch, is also supported, with significant symmetry observed for anomalies in both the upper and lower arches. However, the third hypothesis, positing an influence of gender and malocclusion type on positional anomalies, is not supported by the findings, as no significant differences were observed based on these factors.

This study has several limitations. The relatively small sample size, while adequate for an initial analysis, may not fully represent the broader population, and including more patients could strengthen the significance of the findings. The uneven male-to-female ratio (36/67) introduces a potential bias, as well. Additionally, the retrospective nature of this research and the reliance on digital study models might introduce selection bias, limiting the applicability of the results to other populations. Finally, this study did not explore the potential impact of genetic or environmental factors on the development of positional anomalies, highlighting an area for future research.

Future research should aim to expand the sample size and include a more diverse population to validate our findings. Longitudinal studies could provide insights into the progression of positional anomalies over time and their impact on occlusion and dental health. Additionally, investigating the underlying causes of these anomalies, including genetic predispositions and environmental influences, could lead to more targeted preventive and therapeutic strategies.

## 5. Conclusions

This study highlights the notable prevalence of positional anomalies in second permanent molars, particularly the upper molars (1.7 and 2.7), with a higher incidence of infraocclusion and buccal tilting compared to lower molars. These findings underscore the importance of a closer monitoring of upper molars during routine dental evaluations and call for further research into the underlying factors contributing to these anomalies to support more effective prevention and early intervention strategies.

## Figures and Tables

**Table 1:** Eruption anomalies of second permanent molars.

Eruption Anomaly	Description	Reported Frequency
Ectopic Eruption	Displacement from normal eruption path [14]	1.5% [14]
Impaction	Failure to fully emerge through gingiva [20]	0.06–2.57% [20]
Primary Failure of Eruption	Failure to erupt despite no obstacles [24]	0.06 [24]

**Table 2:** Inclusion and exclusion criteria.

Criteria	Details
Inclusion Criteria	Patients aged 12 or above
	Complete permanent dentition (excluding wisdom teeth)
	Requiring dental treatment
Exclusion Criteria	Extractions or agenesis of permanent teeth
	Previous mobile or fixed orthodontic treatment
	Significant coronal destruction of second permanent molars
	Inadequate scans preventing proper visualization of second permanent molars
	Dental crowns on second permanent molars
	Local or systemic diseases affecting dento-facial growth and development
	Insufficient or incomplete patient records

**Table 3:** Patient distribution by positional anomalies for the upper and lower second permanent molars.

1.7 and 2.7					
PA	2.7—Without PA		2.7—With PA		*p* *
	No.	%	No.	%	
1.7—Without PA	39	75%	9	17.6%	<0.001
1.7—With PA	13	25%	42	82.4%	
**3.7 and 4.7**					
**PA**	**4.7—Without PA**		**4.7—With PA**		0.040
3.7—Without PA	61	80.3%	16	59.3%	
3.7—With PA	15	19.7%	11	40.7%	

PA—positional anomalies; * Fisher’s Exact test.

**Table 4:** Patient distribution by positional anomalies for the right and left second permanent molars.

1.7 and 4.7					
PA	4.7—Without PA		4.7—With PA		*p* *
	No.	%	No.	%	
1.7—Without PA	34	44.7%	14	51.9%	0.654
1.7—With PA	42	55.3%	**13**	48.1%	
**2.7 and 3.7**					
**PA**	**3.7—Without PA**		**3.7—With PA**		0.257
2.7—Without PA	36	46.8%	16	61.5%	
2.7—With PA	41	53.2%	10	38.5%	

PA—positional anomalies; * Fisher’s Exact test.

**Table 5:** Patient distribution according to gender and positional anomalies for second permanent molars.

	Gender/ PA	Female		Male		*p* *
		No.	%	No.	%	
**1.7**	No	30	44.8%	18	50%	0.681
	Yes	37	55.2%	18	50%	
**2.7**	No	34	50.7%	18	50%	1.000
	Yes	33	49.3%	18	50%	
**3.7**	No	46	68.7%	31	86.1%	0.060
	Yes	21	31.3%	5	13.9%	
**4.7**	No	47	70.1%	29	80.6%	0.348
	Yes	20	29.9%	7	19.4%	

PA—positional anomalies; * Fisher’s Exact test.

**Table 6:** Patient distribution according to malocclusion and positional anomalies for second permanent molars.

	Malocclusion/ PA	Class I		Class II/1		Class II/2		Class III		*p* *
		No.	%	No.	%	No.	%	No.	%	
**1.7**	No	19	40.4%	18	54.5%	9	50%	2	40%	0.637
	Yes	28	59.6%	15	45.5%	9	50%	3	60%	
**2.7**	No	22	46.8%	18	54.5%	10	55.6%	2	40%	0.827
	Yes	25	53.2%	15	45.5%	8	44.4%	3	60%	
**3.7**	No	38	80.9%	24	72.7%	11	61.1%	4	80%	0.419
	Yes	9	19.1%	9	27.3%	7	38.9%	1	20%	
**4.7**	No	34	72.3%	23	69.7%	14	77.8%	5	100%	0.622
	Yes	13	27.7%	10	30.3%	4	22.2%	0	0%	

PA—positional anomalies; * Fisher’s Exact test.

## Data Availability

The data presented in this study are available upon request from the corresponding author due to ethical reasons.
